# 

**Published:** 1921-04-02

**Authors:** 


April 2, 1921] [Price Twopence

### The Hospital

The Workers' Newspaper of Administrative Medicine and Institutional Life, Administration, National Insurance and Health.

### CONTENTS

**The American Plan of Hospital Service** ... ... 1

**Crime and Psycho-Analysis** ... ... ... ... 2

**Notes of the Week** ... ... ... ... ... 3

What Will the Approved Societies Do?—Bradford Municipal Hospital—Medical Students and Bradford—Peace Programme of the Red Cross—Venereal Propaganda—Contributors' Certificates—Dangerous Drugs Act Inquiry—"How to Use the New Journalism"—A Statesmanlike Proposal—Red Cross Grants for Building—Mental Hospital Work in Syria—A New Duty for Irish Hospital Secretaries—Another Loss to Clayton Hospital—Belgrade Children's Hospital—What Does a Paying Patient Cost?

**The Peril of the Pocket-Handkerchief** ... ... 6

**Minor Medical Problems** ... ... ... ... 7

Abdominal Pain in Children.

**The Late Sir Felix Semon** ... ... ... ... 8

**A Physician's Anthology** ... ... ... ... 8

**Hospital Neighbourhoods** ... ... ... ... 9

1. Queen Square.

**The Public Well-Being** ... ... ... ... ... 11

Post-War "Nerves"—The Ministry of Health Bill—More about Flogging—A Clinic for Mothers.

**The Hospital World in New Zealand** ... ... 14

The New Health Act.

**Annual Meetings of Hospitals** ... ... ... ... 15

**The Planning of Cottage Hospitals** ... ... ... 16

**The Matrons' and Sisters' Department** ... ... 17

The Pay of the Nurse.—IV. The Ill-effects of the Late Start in Life.

The Value of the Little Hospitals.

**Round the Hospitals** ... ... ... ... 18

**The General Nursing Council for England and Wales** 21

**Scottish Matrons' Association** .. ... ... ... 22

**The Editor's Letter-Box** ... ... .. ... ... 22

Should Nurses Smoke?

**The College of Nursing (Limited by Guarantee)** vi

**The Institutional Worker** ... ... ... ... 1

